# Genome-wide identification and transcriptomic analysis of the MAPK family provides insights into the molecular basis of disease resistance of postharvest eggplant in response to *Botrytis cinerea*

**DOI:** 10.3389/fpls.2025.1680931

**Published:** 2025-09-24

**Authors:** Wenxuan Zhang, Yucheng Ming, Dongchao Ji

**Affiliations:** ^1^ School of Life Sciences and Medicine, Shandong University of Technology, Zibo, China; ^2^ School of Basic Medicine, Qilu Medical University, Zibo, China

**Keywords:** MAPK, eggplant, *Botrytis cinerea*, gene family, transcriptomic analysis

## Abstract

Eggplant (*Solanum melongena*), an important crop for food supply, can suffer from severe gray mold rot caused by *Botrytis cinerea*, resulting in huge postharvest damage every year. Mitogen-activated protein kinase (MAPK) cascades, important to the signal transduction pathway, were identified in many species and proved to be involved in plant growth, development, and immune response, although our knowledge of this cascade in eggplant is scarce. In this work, based on the state-of-art genome sequencing data, the MAPK cascades of eggplant were identified. The result showed that there were 117 *MAP3Ks*, 5 *MAP2Ks*, and 16 *MAPKs* in the eggplant genome. All the proteins possessed traditional MAPK domains. Phylogenetic and collinear analysis showed that eggplant MAPKs was homologous with *Arabidopsis* and tomato. *Cis*-acting element analysis indicated that eggplant MAPKs may participate in defense and stress responsiveness. Meanwhile, transcriptomic analysis of postharvest eggplant after *Botrytis cinerea* infection showed that most of the *MAPK* genes had altered expression; further functional assays indicate that SmMAP3K38 likely operates as a negative regulator of eggplant immunity against *Botrytis cinerea* infection, which provides us new insights into the molecular basis of this important crop in disease resistance to *Botrytis cinerea* and gives us new potential targets for the prevention and control of gray mold.

## 1 Introduction

Eggplant (*Solanum melongena*), a widely grown and consumed vegetable around the world, is the third most cultivated *Solanaceae* crop after tomato (*Solanum lycopersicum*) and potato (*Solanum tuberosum*), especially in India and China, where it is a component in the daily diet (Barchi et al., 2021). With an abundance of vitamins, phenolics, and antioxidants, eggplant provides significant nutritive benefits to human health (Gürbüz et al., 2018). Gray mold is a fungal disease mainly caused by *Botrytis cinerea*, resulting in an annual economic loss of up to US $10–100 billion globally, ranking among the top ten plant fungal diseases (Dean et al., 2012). *Botrytis cinerea* is a necrotrophic fungus that can infect more than 1400 plants, including important economic crops such as tomato, eggplant, strawberry, and grape (Fillinger and Elad, 2016). Because of its convenience and low price, chemical control is still the most widely used method for the prevention and control of gray mold. However, the long-term use of chemical agents has caused problems such as pesticide residues and environmental pollution, which seriously endanger human health (De Miccolis Angelini et al., 2014; Lamichhane et al., 2016). Therefore, locating target genes that regulate plant resistance and susceptibility to pathogens and using targeted genetic manipulation to control gray mold would be an environmentally friendly way to combat this disease (AbuQamar et al., 2017).

Plants have evolved two sets of innate immune mechanisms in the course of long-term interaction with pathogens: pattern-triggered immunity (PTI) and effector triggered immunity (ETI) (Jones and Dangl, 2006). PTI refers to the pattern recognition receptors (PRRs) located on the surface of cells, which recognize conserved MAMPs/DAMPs (Microbe-/Damage-associated molecular patterns), initiate downstream immune responses (including intracellular Ca^2+^ signal transduction, reactive oxygen species (ROS) burst, and mitogen-activated protein kinase (MAPK) cascade activation), and express corresponding immune genes (Yu et al., 2017; Zhang and Zhang, 2022). This layer of defense constitutes the first line. In order to avoid immune processes triggered by conserved molecular patterns, pathogens have evolved effectors that inhibit plant PTI processes to achieve successful plant infection (Jones and Dangl, 2006). Plants further evolved resistance proteins that recognize effectors directly or indirectly, activating the ETI process, which induce local programmed cell death (PCD) or a hypersensitive response (HR) (Boller and He, 2009). After immune receptors recognize MAMPs/DAMPs signals, they typically transmit the signal through a MAPK cascade pathway, which transfers the signal from the receptor to a specific effector to regulate gene expression and cellular activities (Han et al., 2010; Meng and Zhang, 2013). A typical MAPK cascade usually included three components: a MAP kinase kinase kinase (MAP3K/MEKK/MAPKKK/MKKK), a MAP kinase kinase (MAP2K/MKK/MAPKK/MEK), and a MAP kinase (MAPK/MPK) (Zhang and Zhang, 2022). As a close relative of eggplant, there have been several reports on the involvement of the *MAPK* pathway genes in response to *Botrytis cinerea* in tomato. The tomato genome contains 89 MAP3Ks, 5 MAP2Ks, and 16 MAPKs (Wu et al., 2014). Some of them were reported to be involved in the immune response to *Botrytis cinerea*. Transient silencing of *SlMKK2* or *SlMKK4* could decrease the resistance of tomato plants to *Botrytis cinerea*, increase the accumulation of reactive oxygen species, and reduce the expression of defense genes in tomato (Li et al., 2014). The downstream signaling component of MKK was MPK, and silencing of tomato *SlMPK1/2/3/4* could block the defense signaling pathway of tomato fruits and enhance the sensitivity of tomato to *Botrytis cinerea* (Virk et al., 2013; Zheng et al., 2015). After further knocking out *SlMPK3* via CRISPR/Cas9, the *slmpk3* mutant showed reduced activities of immunity-related enzymes, accumulated more reactive oxygen species, and became more susceptible to *Botrytis cinerea* compared with the wild type (Zhang et al., 2018). A recent study revealed that overexpression of *SlMPK8* enhances fruit resistance to *Botrytis cinerea*, whereas the *slmpk8* mutant fruit exhibits increased sensitivity to this pathogen (Deng et al., 2024a). Another result showed that overexpression of *SlMKK2* and *SlMKK4* caused cell death of tomato leaf cells and activation of SlMPK2/3 (Pedley and Martin, 2004). Meanwhile, *in vitro* kinase experiments showed that SlMKK2 and SlMKK4 can phosphorylate SlMPK1/2 (Pedley and Martin, 2004). The above results provide a new way for us to use MAPK as a molecular target for the prevention and control of gray mold.

As the third most cultivated *Solanaceae* crop, little is known about the MAPK family and the role of MAPK in *Botrytis cinerea* infection of eggplant. In this study, through the comprehensive use of a variety of bioinformatics approaches, 117 MAP3Ks, 5 MAP2Ks, and 16 MAPKs were identified in the eggplant genome, and transcriptomic analysis of this family in postharvest eggplant after *Botrytis cinerea* infection provided us new insights and potential targets for the prevention and control of this important fungus.

## 2 Results

### 2.1 Identification of eggplant MAPK family

The identification of the eggplant MAPK family used the local blast programmer from TBtools (Chen et al., 2020). Previously reported MAPK protein sequences from tomato (Kong et al., 2012; Wu et al., 2014) were used as seeds to search for eggplant protein data. As shown in **Table 1**, 117 MAP3Ks, 5 MAP2Ks, and 16 MAPKs were identified in the eggplant genome. MAP3Ks and MAPKs were named according to their location on the chromosome, and MAP2Ks were named based on tomato MAP2Ks. The physicochemical property of the eggplant MAPK family, including protein length (unit: amino acid, aa), molecular weight (unit: Da), theoretical isoelectric point (pI), instability index, aliphatic index, and grand average of hydropathicity (GRAVY), were then analyzed. The results showed that the largest protein was SmMAP3K5, which was composed of 1798 aa, and the smallest protein was SmMAP3K86, consisting of 283 aa.

**Table 1:** List of the eggplant MAPK family identified in the SGN database.

Protein name	SGN locus	Protein length	Molecular weight	Theoretical pI	Instability index	Aliphatic index	GRAVY
SmMAP2K1	SMEL4.1_12g001740.1.01	357	39570.4	5.7	33.77	93.11	-0.068
SmMAP2K2	SMEL4.1_03g034090.1.01	370	40861.67	9.01	61.91	82.24	-0.338
SmMAP2K3	SMEL4.1_03g030750.1.01	469	52392.98	5.3	52.52	95.37	-0.122
SmMAP2K4	SMEL4.1_03g020790.1.01	325	36439.13	8.27	57.53	88.49	-0.228
SmMAP2K5	SMEL4.1_03g022960.1.01	543	60655.23	5.72	37.43	86.74	-0.217
SmMAPK1	SMEL4.1_00g006330.1.01	323	36965.45	6.47	45.73	91.49	-0.234
SmMAPK2	SMEL4.1_01g015420.1.01	373	42977.83	6.02	42.24	85.2	-0.411
SmMAPK3	SMEL4.1_01g024720.1.01	563	64120.31	8.77	40.89	78.13	-0.514
SmMAPK4	SMEL4.1_02g022170.1.01	390	45375.58	8.73	45.55	93.26	-0.284
SmMAPK5	SMEL4.1_04g002760.1.01	583	66119.92	9.28	31.83	81.97	-0.442
SmMAPK6	SMEL4.1_04g021710.1.01	372	42699.36	6.32	43.59	94.35	-0.26
SmMAPK7	SMEL4.1_05g012380.1.01	706	80682.37	7.64	46.37	83.85	-0.444
SmMAPK8	SMEL4.1_05g024580.1.01	376	43136.23	6.6	43.95	90.24	-0.393
SmMAPK9	SMEL4.1_06g004880.1.01	373	42948.28	5.46	36.65	94.13	-0.266
SmMAPK10	SMEL4.1_06g021580.1.01	645	73719.82	6.53	40.68	81.8	-0.47
SmMAPK11	SMEL4.1_07g020660.1.01	619	70279.53	9.18	38.4	78.01	-0.507
SmMAPK12	SMEL4.1_07g021700.1.01	604	68584.94	9.39	33.79	82.04	-0.41
SmMAPK13	SMEL4.1_08g024300.1.01	398	45786.5	6.04	39.96	92.31	-0.245
SmMAPK14	SMEL4.1_10g000210.1.01	503	57536.66	6.67	40	82.25	-0.399
SmMAPK15	SMEL4.1_11g019190.1.01	512	58418.06	7.85	39.2	77.93	-0.418
SmMAPK16	SMEL4.1_12g013130.1.01	369	42523.68	5.06	40.35	94.88	-0.298
SmMAP3K1	SMEL4.1_00g000910.1.01	294	33437.07	5.79	43.34	87.59	-0.369
SmMAP3K2	SMEL4.1_00g001250.1.01	535	59628.65	5.5	41.38	77.12	-0.503
SmMAP3K3	SMEL4.1_00g003090.1.01	757	84698.15	6.54	44.13	75.23	-0.574
SmMAP3K4	SMEL4.1_01g000290.1.01	792	87954.65	5.38	48.9	70.91	-0.61
SmMAP3K5	SMEL4.1_01g002210.1.01	1798	197450.19	6.32	35.37	91.25	-0.17
SmMAP3K6	SMEL4.1_01g002220.1.01	1030	114255.56	5.95	36.24	92.09	-0.123
SmMAP3K7	SMEL4.1_01g002500.1.01	579	65517.58	6.08	50.11	85.87	-0.397
SmMAP3K8	SMEL4.1_01g002900.1.01	529	59059.06	5.79	42.52	81.51	-0.343
SmMAP3K9	SMEL4.1_01g004620.1.01	878	96435.51	6.03	42.89	84.54	-0.164
SmMAP3K10	SMEL4.1_01g011650.1.01	668	73279.35	5.61	53.73	74.3	-0.552
SmMAP3K11	SMEL4.1_01g011790.1.01	981	107424.51	6.19	49.63	80.98	-0.464
SmMAP3K12	SMEL4.1_01g012750.1.01	774	87895.54	5.7	45.88	71.69	-0.64
SmMAP3K13	SMEL4.1_01g014390.1.01	748	83948.15	5.76	47.24	80.01	-0.372
SmMAP3K14	SMEL4.1_01g023970.1.01	712	78418.31	8.36	47.17	73.74	-0.447
SmMAP3K15	SMEL4.1_01g027970.1.01	850	94767.29	6.48	44.05	80.29	-0.256
SmMAP3K16	SMEL4.1_01g035960.1.01	542	61423.41	5.53	43.84	72.32	-0.615
SmMAP3K17	SMEL4.1_01g036370.1.01	567	63475.9	7.2	39.44	85.49	-0.35
SmMAP3K18	SMEL4.1_01g037640.1.01	479	53260.74	8.69	25.27	83.97	-0.314
SmMAP3K19	SMEL4.1_01g038380.1.01	595	67136.6	5.42	42.73	80.94	-0.41
SmMAP3K20	SMEL4.1_02g008660.1.01	358	39706.02	4.7	40.15	83.55	-0.223
SmMAP3K21	SMEL4.1_02g008870.1.01	622	69621.05	9.38	56.98	72.28	-0.592
SmMAP3K22	SMEL4.1_02g010700.1.01	788	87797.39	6.23	55.69	86.7	-0.244
SmMAP3K23	SMEL4.1_02g011700.1.01	461	52436.69	5.94	48.38	82.3	-0.535
SmMAP3K24	SMEL4.1_02g013460.1.01	868	96482.78	5.66	40.89	81.12	-0.273
SmMAP3K25	SMEL4.1_02g014960.1.01	1298	142778.7	5.6	46.52	68.8	-0.645
SmMAP3K26	SMEL4.1_02g016240.1.01	505	57520.08	9.31	49.98	77.82	-0.494
SmMAP3K27	SMEL4.1_02g021390.1.01	815	92376.43	5.75	43.19	87.47	-0.356
SmMAP3K28	SMEL4.1_02g024310.1.01	353	39854.08	7.69	40.85	86.2	-0.218
SmMAP3K29	SMEL4.1_02g026000.1.01	360	39722.22	5.23	38.32	82.89	-0.272
SmMAP3K30	SMEL4.1_02g026010.1.01	358	39717.34	5.08	44.74	83.85	-0.304
SmMAP3K31	SMEL4.1_02g026020.1.01	356	39640.84	5.06	40.08	72.56	-0.381
SmMAP3K32	SMEL4.1_02g026370.1.01	1026	114710.11	5.84	40.63	81.01	-0.273
SmMAP3K33	SMEL4.1_02g027550.1.01	749	83361.84	8.31	46.37	76.57	-0.429
SmMAP3K34	SMEL4.1_03g001510.1.01	484	54946.92	9.4	38.45	76.59	-0.52
SmMAP3K35	SMEL4.1_03g023830.1.01	728	80546.89	5.24	58.24	76.2	-0.594
SmMAP3K36	SMEL4.1_03g025230.1.01	538	60963.04	6.61	36.17	85.54	-0.381
SmMAP3K37	SMEL4.1_03g027050.1.01	850	93804.86	6.03	38.84	86.24	-0.09
SmMAP3K38	SMEL4.1_03g028310.1.01	351	39707.77	8.23	41.91	85.13	-0.275
SmMAP3K39	SMEL4.1_03g028390.1.01	390	43303.52	8.19	35.45	77.97	-0.35
SmMAP3K40	SMEL4.1_03g029050.1.01	424	47022.91	4.57	35.53	79.93	-0.396
SmMAP3K41	SMEL4.1_03g030360.1.01	1029	112458.2	5.18	51.84	78.57	-0.434
SmMAP3K42	SMEL4.1_04g000880.1.01	707	79911.03	5.12	44.65	76.21	-0.477
SmMAP3K43	SMEL4.1_04g002370.1.01	840	91907.43	5.27	37.89	94.46	0.025
SmMAP3K44	SMEL4.1_04g004830.1.01	313	35697.06	5.54	44.55	83.13	-0.208
SmMAP3K45	SMEL4.1_04g005850.1.01	393	44298.8	6.29	31.17	85.85	-0.362
SmMAP3K46	SMEL4.1_04g017310.1.01	956	107070.75	6.35	43.18	84.59	-0.436
SmMAP3K47	SMEL4.1_04g020210.1.01	687	75156.78	8.95	64.29	70.44	-0.561
SmMAP3K48	SMEL4.1_05g003720.1.01	820	90378.18	6.53	37.67	89.37	-0.065
SmMAP3K49	SMEL4.1_05g004710.1.01	819	91513.23	5.65	34.6	90.13	-0.086
SmMAP3K50	SMEL4.1_05g006260.1.01	500	56077.99	5.09	44.8	88.72	-0.298
SmMAP3K51	SMEL4.1_05g006900.1.01	300	33401.02	7.1	34.65	82.93	-0.384
SmMAP3K52	SMEL4.1_05g009320.1.01	362	40806.76	7.54	32.2	83.15	-0.367
SmMAP3K53	SMEL4.1_05g018780.1.01	666	73678.44	8.89	58.38	63.84	-0.679
SmMAP3K54	SMEL4.1_05g019180.1.01	595	67380.21	5.98	55.37	97.6	-0.218
SmMAP3K55	SMEL4.1_05g022020.1.01	767	85054.99	6.45	46.88	73.16	-0.58
SmMAP3K56	SMEL4.1_06g000710.1.01	866	95410.66	6.42	38.33	80.25	-0.298
SmMAP3K57	SMEL4.1_06g000720.1.01	911	100056.89	6.11	34.63	82.52	-0.266
SmMAP3K58	SMEL4.1_06g004680.1.01	829	90489.55	5.8	36.94	85.11	-0.077
SmMAP3K59	SMEL4.1_06g005010.1.01	1006	108251.53	9.28	58.92	74.2	-0.396
SmMAP3K60	SMEL4.1_06g012070.1.01	893	96035.11	9.43	60.6	66.34	-0.557
SmMAP3K61	SMEL4.1_06g021080.1.01	420	47018.17	5.05	41.95	77.98	-0.467
SmMAP3K62	SMEL4.1_06g021570.1.01	1014	111036.72	5.78	50.03	78.23	-0.46
SmMAP3K63	SMEL4.1_06g023040.1.01	464	52333.31	8.91	39.89	84.01	-0.326
SmMAP3K64	SMEL4.1_06g023480.1.01	651	72909.32	4.96	48.34	80.15	-0.541
SmMAP3K65	SMEL4.1_06g024970.1.01	536	61117.9	5.85	31.29	82.33	-0.391
SmMAP3K66	SMEL4.1_06g028700.1.01	647	73934.62	5.9	45.47	76.83	-0.52
SmMAP3K67	SMEL4.1_07g000600.1.01	1239	138267.45	5	45.59	68.9	-0.662
SmMAP3K68	SMEL4.1_07g002610.1.01	1422	153161.15	5.31	40.95	75.51	-0.433
SmMAP3K69	SMEL4.1_07g011540.1.01	428	47069.01	6.41	37.42	75.23	-0.479
SmMAP3K70	SMEL4.1_07g012120.1.01	318	35482.46	7.78	32.83	82.23	-0.399
SmMAP3K71	SMEL4.1_07g012220.1.01	885	96959.49	5.77	43.66	79.51	-0.209
SmMAP3K72	SMEL4.1_07g016560.1.01	334	37023.83	5.47	56.47	82.07	-0.344
SmMAP3K73	SMEL4.1_07g016570.1.01	333	37219.2	5.39	53.96	82.88	-0.396
SmMAP3K74	SMEL4.1_07g016580.1.01	555	61090.22	5.6	51.24	81.39	-0.34
SmMAP3K75	SMEL4.1_07g016590.1.01	332	36690.36	5.36	57.02	79.04	-0.409
SmMAP3K76	SMEL4.1_07g016600.1.01	381	42478.97	5.18	59.26	80.13	-0.444
SmMAP3K77	SMEL4.1_07g016610.1.01	390	43368.98	5.37	45.48	80	-0.345
SmMAP3K78	SMEL4.1_07g017710.1.01	603	66114.34	5.62	40.49	82.24	-0.371
SmMAP3K79	SMEL4.1_07g019430.1.01	826	92705.68	6.16	57.82	83.72	-0.391
SmMAP3K80	SMEL4.1_07g020530.1.01	470	53343.24	6.27	51.29	89.62	-0.353
SmMAP3K81	SMEL4.1_07g023150.1.01	843	92227.57	5.51	37.69	89.18	-0.078
SmMAP3K82	SMEL4.1_07g024490.1.01	467	52021.9	4.41	42.4	90.96	-0.029
SmMAP3K83	SMEL4.1_07g024910.1.01	307	35458.17	5.29	32.06	85.31	-0.506
SmMAP3K84	SMEL4.1_08g000240.1.01	520	58411.24	5.73	41.96	77.81	-0.453
SmMAP3K85	SMEL4.1_08g001230.1.01	741	82858.55	6.19	46.92	67.09	-0.691
SmMAP3K86	SMEL4.1_08g011840.1.01	283	32781.78	5.82	52.83	97.49	-0.243
SmMAP3K87	SMEL4.1_08g024620.1.01	877	94251.83	9.25	62.46	66.81	-0.481
SmMAP3K88	SMEL4.1_08g025400.1.01	742	83124.96	6.78	50.51	77.37	-0.487
SmMAP3K89	SMEL4.1_08g025500.1.01	591	66847.48	5.19	46.32	88.56	-0.363
SmMAP3K90	SMEL4.1_09g001250.1.01	878	96798.93	5.85	48.84	83.96	-0.307
SmMAP3K91	SMEL4.1_09g007090.1.01	529	59621.91	6.04	36.62	84.61	-0.44
SmMAP3K92	SMEL4.1_09g010060.1.01	357	40106.23	7.09	41.36	92.27	-0.201
SmMAP3K93	SMEL4.1_09g010100.1.01	596	68369.44	5.64	46.54	80.64	-0.476
SmMAP3K94	SMEL4.1_09g010930.1.01	888	97206.73	5.69	42.59	80.01	-0.207
SmMAP3K95	SMEL4.1_09g019210.1.01	731	82920.35	5.01	53.48	73.86	-0.621
SmMAP3K96	SMEL4.1_10g010500.1.01	812	90741.61	6.99	32.04	86.51	-0.118
SmMAP3K97	SMEL4.1_10g015030.1.01	435	49096.16	8.16	37.58	83.63	-0.464
SmMAP3K98	SMEL4.1_10g019780.1.01	947	104217.66	6	39.21	93.9	-0.151
SmMAP3K99	SMEL4.1_10g020400.1.01	466	53221.03	8.85	33.23	82.66	-0.534
SmMAP3K100	SMEL4.1_10g022610.1.01	849	94326.31	5.94	52.68	84.51	-0.307
SmMAP3K101	SMEL4.1_10g026030.1.01	524	59392.73	6.03	33.19	83.13	-0.51
SmMAP3K102	SMEL4.1_10g027240.1.01	602	66978.89	5.42	44.08	80.78	-0.406
SmMAP3K103	SMEL4.1_11g004390.1.01	608	66586.18	9.2	54.89	70.86	-0.533
SmMAP3K104	SMEL4.1_11g004820.1.01	593	66200.53	5.51	38.74	81.06	-0.381
SmMAP3K105	SMEL4.1_11g012290.1.01	510	57165.26	5.21	40.03	87	-0.324
SmMAP3K106	SMEL4.1_11g023790.1.01	376	42894.51	9.14	53.32	78.86	-0.377
SmMAP3K107	SMEL4.1_11g025330.1.01	1445	159140.42	6.16	45.31	91.07	-0.315
SmMAP3K108	SMEL4.1_11g026050.1.01	580	64563.28	5.52	35.04	78.29	-0.411
SmMAP3K109	SMEL4.1_11g027360.1.01	368	42174.04	9.03	51.22	82.42	-0.296
SmMAP3K110	SMEL4.1_12g002090.1.01	405	44876.35	6.32	31.15	86.17	-0.18
SmMAP3K111	SMEL4.1_12g002850.1.01	553	61920.11	5.5	40.47	80.42	-0.37
SmMAP3K112	SMEL4.1_12g003140.1.01	596	67088.61	5.86	51.17	86.19	-0.377
SmMAP3K113	SMEL4.1_12g003870.1.01	847	93940.44	5.44	35.97	81.89	-0.188
SmMAP3K114	SMEL4.1_12g006000.1.01	499	56229.19	5.71	37.16	83.65	-0.374
SmMAP3K115	SMEL4.1_12g010660.1.01	541	60972.21	5.88	37.3	77.67	-0.538
SmMAP3K116	SMEL4.1_12g011580.1.01	571	63882.2	6.62	39.74	82.29	-0.386
SmMAP3K117	SMEL4.1_12g012720.1.01	837	91980.58	6.39	35.59	82.75	-0.125

### 2.2 Phylogenetic analysis of the eggplant MAPK family

**Figure 1:**
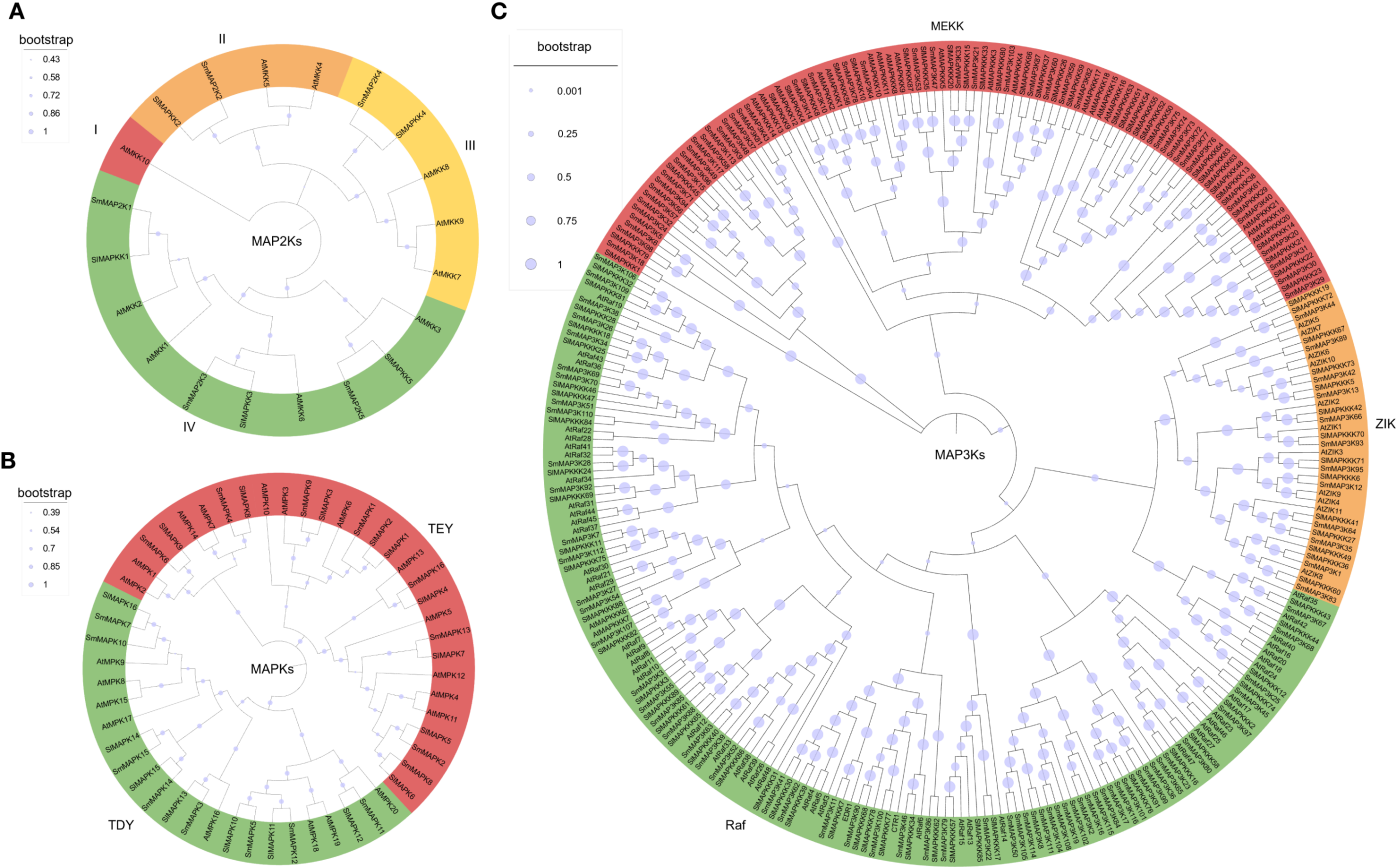
Phylogenetic analysis of the eggplant MAPK family. The phylogenetic analysis of eggplant MAP2Ks **(A)**, MAPKs **(B)**, and MAP3Ks **(C)**.

To explore the relationships among eggplant, tomato, and *Arabidopsis* MAPKs, the phylogenetic analyses using these protein sequences were conducted. The results showed that the three species of MAP2Ks could be divided into four groups (group I to IV, **Figure 1A**). In group I, there was only one member, *Arabidopsis* AtMKK10, while Group II to IV had 4, 5, and 10 eggplant, tomato, and *Arabidopsis* members respectively. This result indicates that eggplant MAP2Ks and tomato share a close phylogenetic relationship. As for MAPKs, all the eggplant, tomato, and *Arabidopsis* MAPKs were divided into two groups according to their conserved protein domain, TEY (Thr-Glu-Tyr) and TDY (Thr-Asp-Tyr) (**Figure 1B**). MAP3Ks were the largest MAPKs. The phylogenetic analysis showed that eggplant MAP3Ks also had three subgroups: MEKK, ZIK, and Raf (**Figure 1C**). Consistent with previous reports in tomato, the Raf subfamily constitutes the largest proportion of MAP3K members.

### 2.3 The gene collinearity of the eggplant MAPK family.

**Figure 2:**
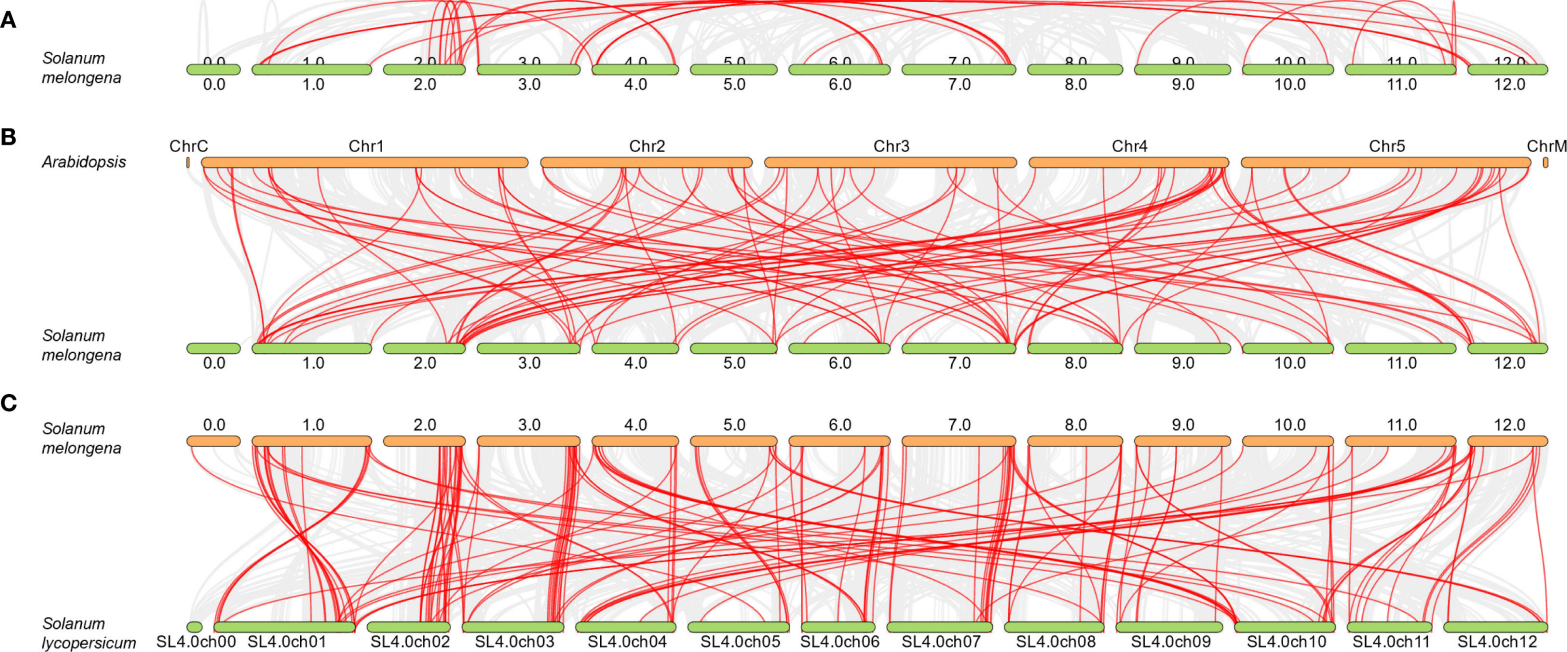
Gene collinearity analysis of the eggplant *MAPK* genes. Collinearity analysis of MAPK genes among eggplant itself **(A)**, eggplant versus *Arabidopsis* **(B)**, and eggplant versus tomato **(C)**.

To gain a deeper insight into the genetic duplication occurrences within the eggplant *MAPKs* gene family, their collinearity was examined utilizing the One Step MCScanX tool available in TBtools. The analysis revealed that there were 50 pairs of *SmMAPK* genes exhibiting collinearity, indicating the presence of intraspecific duplication events (**Figure 2A**, **Supplementary Table S1**). Additionally, a detailed investigation was conducted to explore the collinearity between eggplant *MAPKs* genes and those of *Arabidopsis* and tomato. This additional analysis aimed to provide a more comprehensive understanding of the genetic relationships and potential duplication events across these species. This led to the identification of 94 and 156 pairs of collinear genes between eggplant and those of *Arabidopsis* and tomato, respectively (**Figures 2B, C**, **Supplementary Table S1**). These findings hint at the possibility of interspecific duplication events having taken place during their evolutionary history.

### 2.4 Chromosome location of the eggplant MAPK family

**Figure 3:**
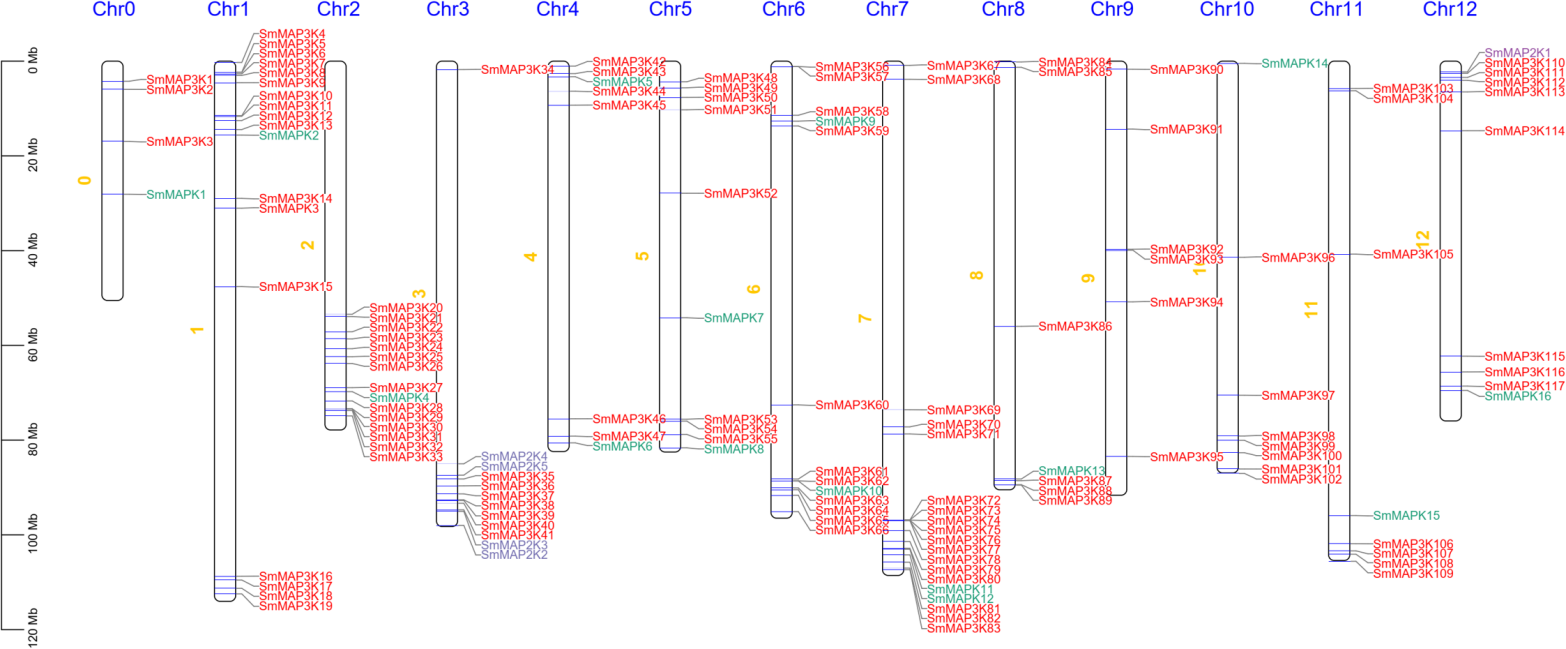
The chromosome location of the eggplant MAPK family. The *MAP3Ks*, *MAP2Ks*, and *MAPKs* were marked with red, blue, and green respectively. Scale bar indicates chromosome length. Scar bar = 20 Mb.

The 138 eggplant *MAPKs* genes were anchored on the eggplant chromosomes, as shown in **Figure 3**. Among them, SmMAPKs genes were distributed across all chromosomes except chromosomes 3 and 9, while *SmMAP2Ks* genes were mainly distributed on chromosomes 3 and 12. Members of the eggplant *MAP3K* family were conspicuously clustered along the chromosomes.

### 2.5 Gene structure and protein domain analysis of the eggplant MAPK family

Using the Batch-CD search tool from NCBI, the conserved protein domains of eggplant MAPKs were acquired. As shown in **Supplementary Figure S1** and **Supplementary Table S2**, the SmMAPKs mainly included STKc_TEY_MAPK or STKc_TDY_MAPK domains, the SmMAP2Ks mainly consisted of PKc_MAPKK_plant_like domains, and the SmMAP3Ks mainly contained STKc_ MAPKKK, STKc_MEKK1_plant, or STKc_MAP3K_like domains, which further confirmed the accuracy of these identified proteins. Otherwise, the distribution of these domains was varied in different proteins. They could be located in the N-terminal, middle space, or C-terminal. In addition, with the help of gene annotation file, the gene structures of eggplant *MAPKs* were acquired and visualized. Most of the eggplant *MAPK* genes owned one or more introns, while some of them had a complete CDS that was not interrupted by introns (**Supplementary Figure S1**).

### 2.6 *Cis*-acting element analysis of the eggplant MAPK family

MAPKs were reported to be involved in plant growth, development, and stress responses (Meng and Zhang, 2013; Xu and Zhang, 2015; Zhang and Zhang, 2022). *Cis*-acting elements can give us information about the possible functions of genes. In order to preliminarily analyze whether eggplant MAPKs also participated in the above process, the promoters of these 138 genes were extracted by TBtools and analyzed by PlantCARE to test the *cis*-acting elements. The results showed that all the promoters of eggplant *MAPKs* genes held diverse *cis*-acting elements, including light response, hormone response, and defense and stress response, indicating that eggplant *MAPK* genes might participate in these biological progresses (**Supplementary Figure S2**, **Supplementary Table S3**).

### 2.7 Transcriptomic analysis of the eggplant *MAPK* family genes in response to *Botrytis cinerea* infection

**Figure 4:**
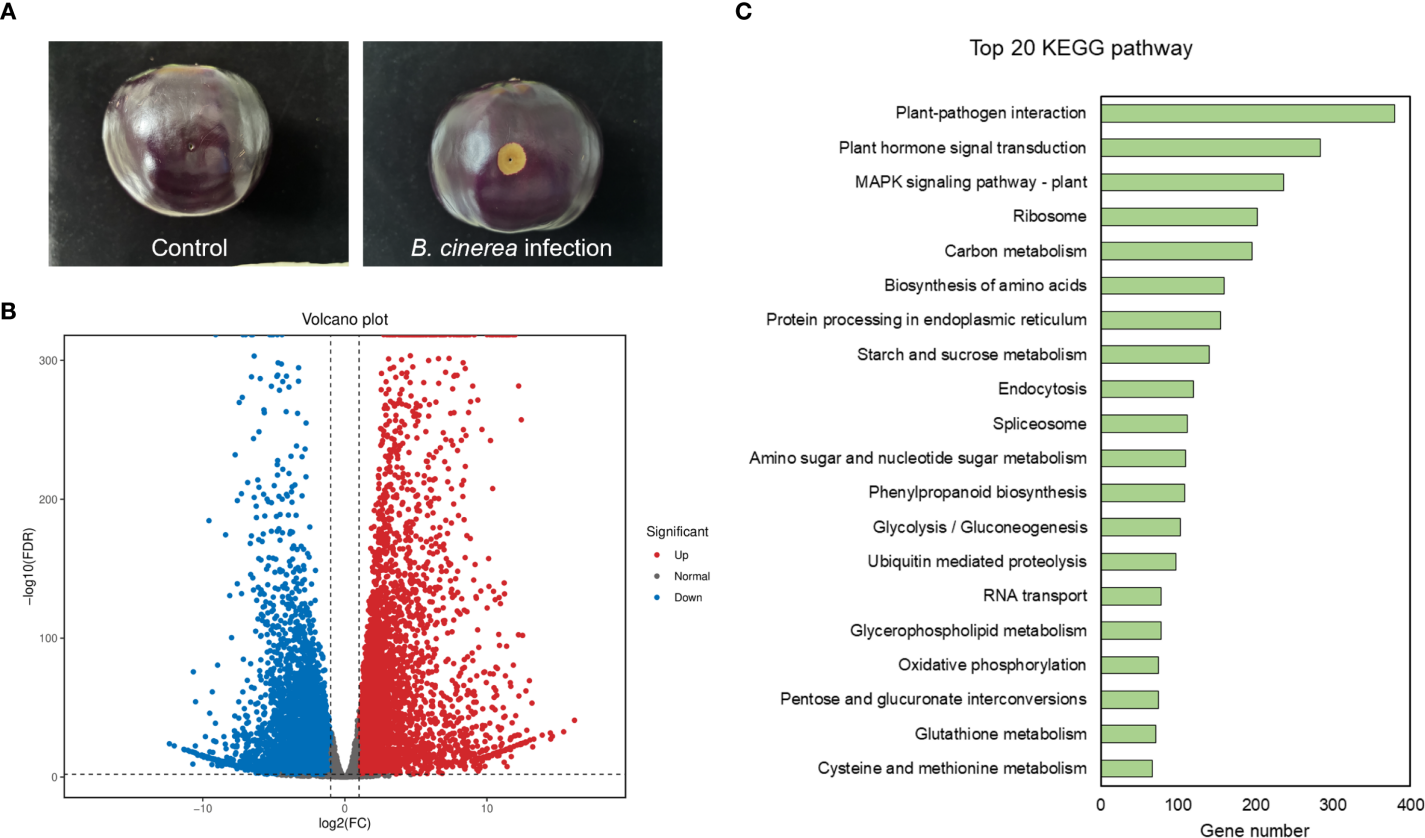
Transcriptomic analysis of the eggplant genes in response to *Botrytis cinerea* infection. **(A)** The representative images depict the eggplant samples used in the transcriptome experiment. **(B)** Volcano plot of up- and down-regulated genes in eggplant before and after infection by *Botrytis cinerea*. Red indicates up-regulation, blue indicates down-regulation, and gray indicates no significant difference. **(C)** KEGG pathway analysis of the DEGs. Categories are ranked according to the number of genes obtained by clustering.

*Cis*-acting elements analysis indicated that eggplant *MAPK* genes might participate in defense response. Gray mold is a severe disease of eggplant caused by *Botrytis cinerea*. In order to further explore the function of *MAPKs* family genes in eggplant, transcriptome sequencing technology was used to detect the gene expression levels of *MAPK* family genes before and after the fruit was infected with *Botrytis cinerea* (**Figure 4A**). The results were as follows.

To validate the reliability of our transcriptomic experiment, we first mined the literature for well-established disease-resistance marker genes in tomato (*PR1a1*, *GRAS4.1*, *WRKY28*, *PTI5*, *LRR22*, and *GRAS2*) (Nguyen et al., 2010; Taylor et al., 2012; Harel et al., 2014; Topalović et al., 2020). Their eggplant orthologs were then retrieved by reciprocal BLAST searches. Expression profiling confirmed that these orthologous genes were markedly up-regulated following *Botrytis cinerea* infection, corroborating the robustness of our experimental treatment (**Supplementary Figure S3**). Then, differential expression analysis revealed that there were 10496 differentially expressed genes (DEGs) between the *Botrytis cinerea* infection group and control group. After *Botrytis cinerea* infection, 5531 genes were down-regulated and 4965 genes were up-regulated (**Figure 4B**). For functional classification of the differentially expressed genes (DEGs), KEGG pathway enrichment analysis was performed (**Figure 4C**). Interestingly, 236 DEGs were enriched in the MAPK signaling pathway-plant term of KEGG, indicating that MAPK signaling pathway genes might play important roles in eggplant to *Botrytis cinerea* infection. The gene expression of the 138 newly identified eggplant *MAPKs* were analyzed by transcriptomic sequencing. As shown in **Supplementary Figure S4** and **Supplementary Table S4**, most of the genes were up-regulated and some of the genes were down-regulated after *Botrytis cinerea* infection. However, 26 genes had no available data due to the limitation of sequencing technology or the low expression level.

### 2.8 SmMAP3K38 is involved in the response of eggplant to *Botrytis cinerea*

**Figure 5:**
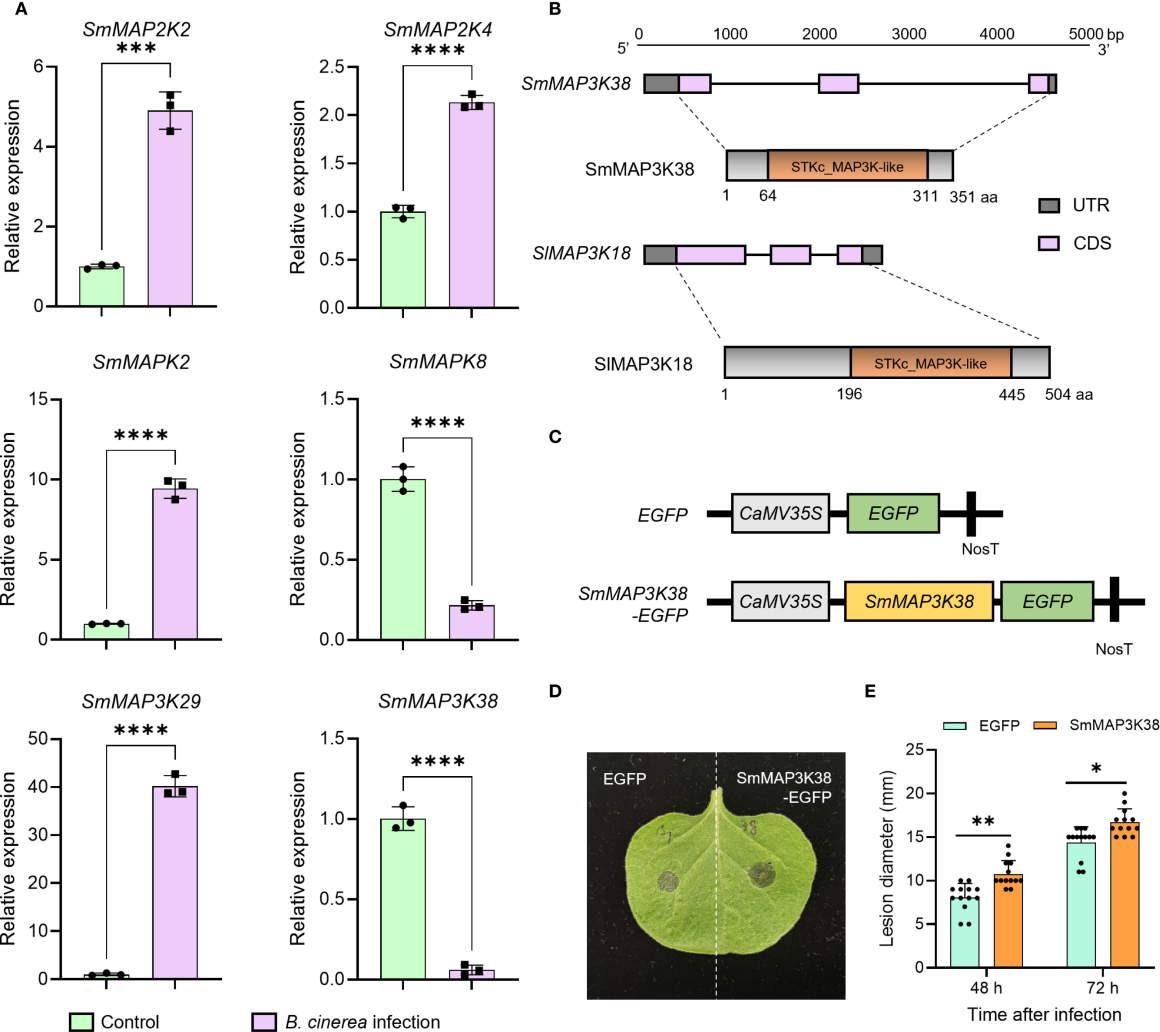
SmMAP3K38 is involved in response to *Botrytis cinerea* infection. **(A)** qPCR confirms the expression trends of six MAPK-component genes (*SmMAP2K2*, *SmMAP2K4*, *SmMAPK2*, *SmMAPK8*, *SmMAP3K29* and *SmMAP3K38*) identified in the transcriptome. **(B)** The comparison of gene and protein structure between SmMAP3K38 and SlMAP3K18. **(C)** Schematic diagram of the *SmMAP3K38* expression vector construction. **(D)** Representative photograph of gray mold lesions on tobacco leaves 48 h after inoculation with *Botrytis cinerea* spore suspension following transient expression of *SmMAP3K38*. **(E)** Statistical analysis of gray mold lesion diameters on tobacco leaves transiently expressing *SmMAP3K38*. Data were mean values ± SD. Asterisks indicate significant difference (*P < 0.05, **P < 0.01, ***P < 0.001, ****P < 0.0001; Student’s t-test).

Transcriptome profiling revealed that MAPK cascade components across multiple tiers displayed marked expression shifts following *Botrytis cinerea* challenge. To corroborate these findings, we selected the *SmMAP2Ks*, *SmMAPKs*, and *SmMAP3Ks* exhibiting the most pronounced transcriptional changes and re-examined their transcript abundance by qPCR. The resulting expression patterns were fully concordant with the RNA-seq data, providing independent confirmation of the reliability of our transcriptomic dataset (**Figure 5A**). As the first signaling component of the MAPK cascade, MAP3K usually receives upstream pathogen signals directly and plays a key role within the MAPK module. Our previous work demonstrated that tomato SlMAP3K18 perceives upstream immune cues and precisely modulates the fruit’s response to *Botrytis cinerea* (Ji et al., 2023). The present transcriptomic data reveal *SmMAP3K38* as the MAPK-family gene exhibiting the greatest down-regulated expression change. It belongs to the Raf clade, clusters closely with SlMAP3K18 in phylogenetic analysis, and shares similar genes and protein architectures (**Figure 5B**). SmMAP3K38 is therefore highly likely to participate in eggplant’s defense against *Botrytis cinerea*, and its expression profile suggests it acts as a negative regulator. To test this hypothesis, we constructed expression vectors to express EGFP (control) and SmMAP3K38-EGFP (experimental) in the model plant *Nicotiana benthamiana* (**Figure 5C**), followed by inoculation with *Botrytis cinerea* spore suspension. Lesion diameters were significantly larger in SmMAP3K38-expressing leaves than in controls (**Figures 5D, E**), supporting our hypothesis. These results indicate that SmMAP3K38 serves as a negative regulator of the eggplant defense response to *Botrytis cinerea*.

## 3 Methods

### 3.1 Plant and fungi materials

Eggplant fruit (*Solanum melongena* var. *esculentum*) was purchased in Zibo City, Shandong Province Zhangdian District supermarket. The *Botrytis cinerea* was isolated from naturally infected strawberry fruits and preserved in the laboratory. The *Botrytis cinerea* was routinely maintained on potato dextrose agar (PDA) and incubated at 23 ± 2°C for 7–10 days until profuse sporulation. Conidia were gently dislodged by flooding each plate with sterile distilled water, and the resulting suspension was filtered through four layers of sterile cheesecloth to remove mycelial debris. A hemocytometer count adjusted the final spore density to 2 × 10^5^ spores per mL.

### 3.2 Gene family identification and phylogenetic analysis

The eggplant and tomato protein sequences were extracted from SGN (Sol Genomics Network, https://solgenomics.net/) (Fernandez-Pozo et al., 2015). The tomato MAPK, MAP2K, and MAP3K protein sequences were set as reference sequences to conduct blast analysis in eggplant protein database using TBtools. The results were filtered by E-value=0. The *Arabidopsis* MAPK protein sequences were downloaded from TAIR (https://www.arabidopsis.org/) (Berardini et al., 2015). All candidate eggplant sequences were submitted to the NCBI Batch-CD database (https://www.ncbi.nlm.nih.gov/Structure/bwrpsb/bwrpsb.cgi)?. Among the returned results (Batch CD hitdata-full), sequences with clear MAPK, MAP2K, or MAP3K annotations were selected for subsequent analysis.

Then, these sequences were sent to ClustalW to align. The neighbor-joining phylogenetic tree were constructed by MEGA, version 11 (Tamura et al., 2021) (State College, PA, USA), with 1,000 bootstrap replicates, a pairwise deletion, and a Poisson model. The tree file was optimized by iTOL, version 6 (Letunic and Bork, 2024) (https://itol.embl.de/) (Heidelberg, Germany).

### 3.3 Chromosome location

The chromosomal locations of the *MAPK*, *MAP2K*, and *MAP3K* genes in eggplant were retrieved from the SGN and visualized using TBtools.

### 3.4 Gene structure and protein domain analysis

Protein domain analysis was performed by submitting the amino acid sequences to the Batch CD-Search tool under default parameters, with only hits meeting the significance threshold (E-value < 1×10^−10^) retained for further analysis.

For gene structure analysis, the corresponding gene annotation files were retrieved from the Solanaceae Genomics Network (SGN) database and subsequently visualized using the Gene Structure View module in TBtools.

### 3.5 *Cis*-acting element analysis

The 2,000 base pair (bp) region located upstream of the coding sequence start sites for *SmMAPK*, *SmMAP2K*, and *SmMAP3K* was extracted from the eggplant genome utilizing the GXF Sequence Extract function within TBtools. Following this extraction, the sequence was subjected to analysis using PlantCARE (available at http://bioinformatics.psb.ugent.be/webtools/plantcare/html/) (Lescot, 2002), with the aim of identifying *cis*-acting elements under standard parameters. The outcomes of this analysis were subsequently visualized employing the Simple BioSequence Viewer feature of TBtools version 1.108.

### 3.6 Gene collinearity analysis

The genomic data of eggplant, tomato, and *Arabidopsis* were downloaded from SGN and TAIR. These sequence data were used to conduct gene collinearity analysis using TBtools.

### 3.7 Transcriptomic analysis

For inoculation, the eggplant fruits were washed with sterile water three times, then disinfected with 2% sodium hypochlorite and washed again with sterile water three times. The eggplants were subjected to a standardized incision process: a sterilized scalpel created wounds on the fruits’ equatorial region, each 2 mm in width and 5 mm in depth, prior to inoculation. Each incision received a 5 μL application of a spore suspension, with a concentration of 2 × 10^5^ spores per mL. For comparison, a set of fruits was treated with water and served as the control group. All fruits were then placed in plastic containers, maintaining a high humidity level of approximately 95%, and were kept at an ambient temperature of 23 ± 2°C for a duration of four days. Both the control and *Botrytis cinerea* treatment groups comprised three biological replicates.

For transcriptome sequencing, total RNA were extracted and sent to Biomarker Technologies (Qingdao, China). Raw FASTQ reads were trimmed with in-house Perl scripts to remove adapters, poly-N tails, and low-quality bases; adapter- and quality-filtered reads were discarded. High-quality reads were aligned to the reference genome with HISAT2, keeping only uniquely mapped reads with ≤1 mismatch. Differential expression between groups was determined with DESeq2 (negative-binomial model, FDR < 0.01, |log2 Fold Change (log2FC)| ≥ 1). Statistical enrichment of differentially expressed genes in KEGG pathways was assessed with KOBAS (Mao et al., 2005) and clusterProfiler.

### 3.8 RT-qPCR

Total RNA was reverse-transcribed into first-strand cDNA using the Hifair^®^ III 1st Strand cDNA Synthesis SuperMix for qPCR (gDNA digester plus) according to the manufacturer’s instructions (Yeasen Biotech, Shanghai, China). RT-qPCR was performed on a LightCycler 480 Instrument II platform (Roche) with Hieff UNICON Universal Blue SYBR Green Master Mix (Yeasen) following the manufacturer’s instructions. Primer pairs specific to each target (**Supplementary Table S5**) were designed with TBtools, and *SmACTIN* (Deng et al., 2024b) served as the reference gene. Data are presented as means ± SD of three replicates.

### 3.9 Gene clone and pathogen inoculation

*SmMAP3K38* CDS was amplified from eggplant fruit cDNA and ligated into the pEAQ-EGFP backbone downstream of the CaMV 35S promoter and upstream of the NOS terminator. Sequence-verified plasmids were introduced into *Agrobacterium tumefaciens* GV3101. Transformed bacteria were cultured overnight at 28°C in LB supplemented with kanamycin and rifampicin and resuspended in infiltration buffer (10 mM MES, pH 5.7, 10 mM MgCl_2_, 200 µM acetosyringone) to an OD_600_ of 0.5. After 2 h incubation, the suspension was pressure-infiltrated into the abaxial surface of 4-week-old *Nicotiana benthamiana* leaves with a needleless 1 mL syringe. Leaves were harvested 48 h post-infiltration for pathogen challenge, following the protocol described previously (Ji et al., 2023). The primers used in this study are listed in **Supplementary Table S5**.

## 4 Discussion

The present study provides a comprehensive genome-wide identification and transcriptomic analysis of the MAPK family genes in eggplant, elucidating their potential roles in the molecular basis of disease resistance against *Botrytis cinerea* postharvest. This investigation offers valuable insights into the complex regulatory mechanisms underlying the defense responses in eggplant, a crucial horticultural crop.

Through a systematic genome-wide approach, we identified a total of 138 MAPK family genes in eggplant. The uneven distribution of these genes across the chromosomes suggests the occurrence of gene duplication events, which may have contributed to the functional diversification of the MAPK family in eggplant. The conserved domain analysis revealed a high degree of evolutionary conservation among MAPK family genes, implying that they may share similar functions across different plant species. This conservation is essential for understanding the fundamental roles of MAPKs in plant stress and pathogen responses and provides a basis for comparative studies with other species.

The transcriptomic analysis unveiled dynamic expression patterns of MAPK family genes during the postharvest infection of eggplant by *Botrytis cinerea*. Transcriptome analysis revealed that the majority of genes exhibited upregulation trends, while a subset showed downregulation. Excluding genes with undetectable expression levels, all *SmMAP2K* genes were upregulated, whereas some *SmMAPK* genes displayed minimal expression changes. Notably, *SmMAP3K* genes demonstrated diverse expression patterns. Of particular interest, following *Botrytis cinerea* infection, *SmMAP3K29* showed the highest upregulation fold-change while *SmMAP3K38* exhibited the most significant downregulation. As the primary signaling components in the MAPK cascade, MAP3Ks may be associated with the specificity of infection signal recognition. This differential expression pattern highlights the multifaceted functions of MAPK family genes in the resistance of eggplant to *Botrytis cinerea*, encompassing early pathogen recognition, signal transduction, and downstream defense response regulation.

In our previous work, we demonstrated that tomato SlMAP3K18 acts as a molecular bridge linking receptor-like kinases to downstream MAP2Ks and thereby modulates the fruit’s response to *Botrytis cinerea* (Ji et al., 2023). The eggplant ortholog SmMAP3K38 shares extensive sequence and structural similarity with SlMAP3K18, and pathogen challenge assays reveal that SmMAP3K38 likewise functions as a negative regulator of immunity against *Botrytis cinerea*. These findings suggest that this clade of MAP3Ks performs a conserved role across *Solanaceous* species. Nevertheless, functional divergence is evident. *SlMAP3K18* is transcriptionally up-regulated upon *Botrytis cinerea* infection, yet transient silencing of *SlMAP3K18* in tomato does not alter disease susceptibility, implying that its primary contribution lies in signal transmission via phosphorylation rather than in modulating transcript abundance. MAPK cascades operate through sequential phosphorylation events, and their output is likely conditioned by the abundance of each signaling component, which are themselves subject to transcriptional control. Our current data indicate that transcriptional regulation of *SmMAP3K38* contributes—at least in part—to its functional output. Comparable scenarios have been reported for other MAPK constituents. *Botrytis cinerea* infection induces *SlMKK2* (*SlMAP2K2*) and *SlMKK4* (*SlMAP2K4*) expression in tomato and transient silencing of either gene enhances susceptibility, while over-expression reduces it (Li et al., 2014). Silencing or knockout of *SlMPK1/2/3/4/8* renders tomato markedly more susceptible to *Botrytis cinerea* infection (Virk et al., 2013; Zheng et al., 2015; Zhang et al., 2018; Deng et al., 2024a). Collectively, these results underscore a nuanced interplay between transcriptional control and post-translational phosphorylation in shaping the strength and specificity of MAPK-mediated defense responses.

Based on the expression patterns and existing literature, we propose that MAPK family genes may function through multiple molecular mechanisms in the resistance of eggplant to *Botrytis cinerea*. Initially, the MAPK signaling pathway is likely involved in the recognition of PAMPs by pattern recognition receptors (PRRs) on the plant cell surface, triggering early immune responses (Yu et al., 2017; Zhang and Zhang, 2022; Ji et al., 2023). This process may activate a cascade of downstream signaling components, including calcium ion signaling, transcription factors, and hormone signaling pathways. The eggplant MAPK family genes may participate in and regulate the synthesis and signaling of plant hormones such as salicylic acid (SA), jasmonic acid (JA), and auxin, thereby influencing the plant’s defense responses, as shown in the *cis*-acting element analysis of this family. Additionally, MAPK family genes may be involved in the signaling of cell wall-related pathways, reinforcing the structural integrity of the cell wall to restrict pathogen invasion and spread (Meng and Zhang, 2013). This multi-layered regulatory mechanism provides significant clues for understanding the molecular basis of disease resistance in eggplant.

This study significantly advances our understanding of the molecular mechanisms underlying postharvest disease resistance in eggplant by providing a detailed characterization of the MAPK family genes and their potential roles in defense against *Botrytis cinerea*. The findings not only offer valuable genetic resources for developing new disease-resistant strategies but also contribute to the broader field of plant pathology and molecular biology. Future research should focus on functional validation of these MAPK family genes using techniques such as CRISPR/Cas9 gene editing, overexpression analyses, and single-cell and spatial RNA sequencing (Deng et al., 2024a; Dong et al., 2024; Li et al., 2024) to elucidate their specific roles and molecular mechanisms in disease resistance. Additionally, integrating protein-protein interaction network analyses and metabolomics studies could provide a holistic understanding of the complex regulatory networks involving the MAPK signaling pathway in plant defense responses.

## Data Availability

The raw sequence data reported in this paper have been deposited in the Genome Sequence Archive (Chen et al., 2021) in National Genomics Data Center (CNCB-NGDC Members and Partners et al., 2025), China National Center for Bioinformation/Beijing Institute of Genomics, Chinese Academy of Sciences (GSA: CRA029243) that are publicly accessible at https://ngdc.cncb.ac.cn/gsa.
