# Supplementary material for: Genome-wide identification and transcriptomic analysis of the MAPK family provides insights into the molecular basis of disease resistance of postharvest eggplant in response to Botrytis cinerea
Source: Front Plant Sci. 2025 Sep 24;16:1680931. doi: 10.3389/fpls.2025.1680931 (PMC12504102; doi:10.3389/fpls.2025.1680931)

**Supplementary Figures and Figure legends**

Figure S1 Gene structure and protein domain analysis of the eggplant MAPK family. Typical MAPK domains are color-mapped on each protein. Scale: amino acid (aa). Untranslated region (UTR) and coding sequence (CDS) are annotated on the gene model. Scale: base pair (bp). Lines indicate introns.

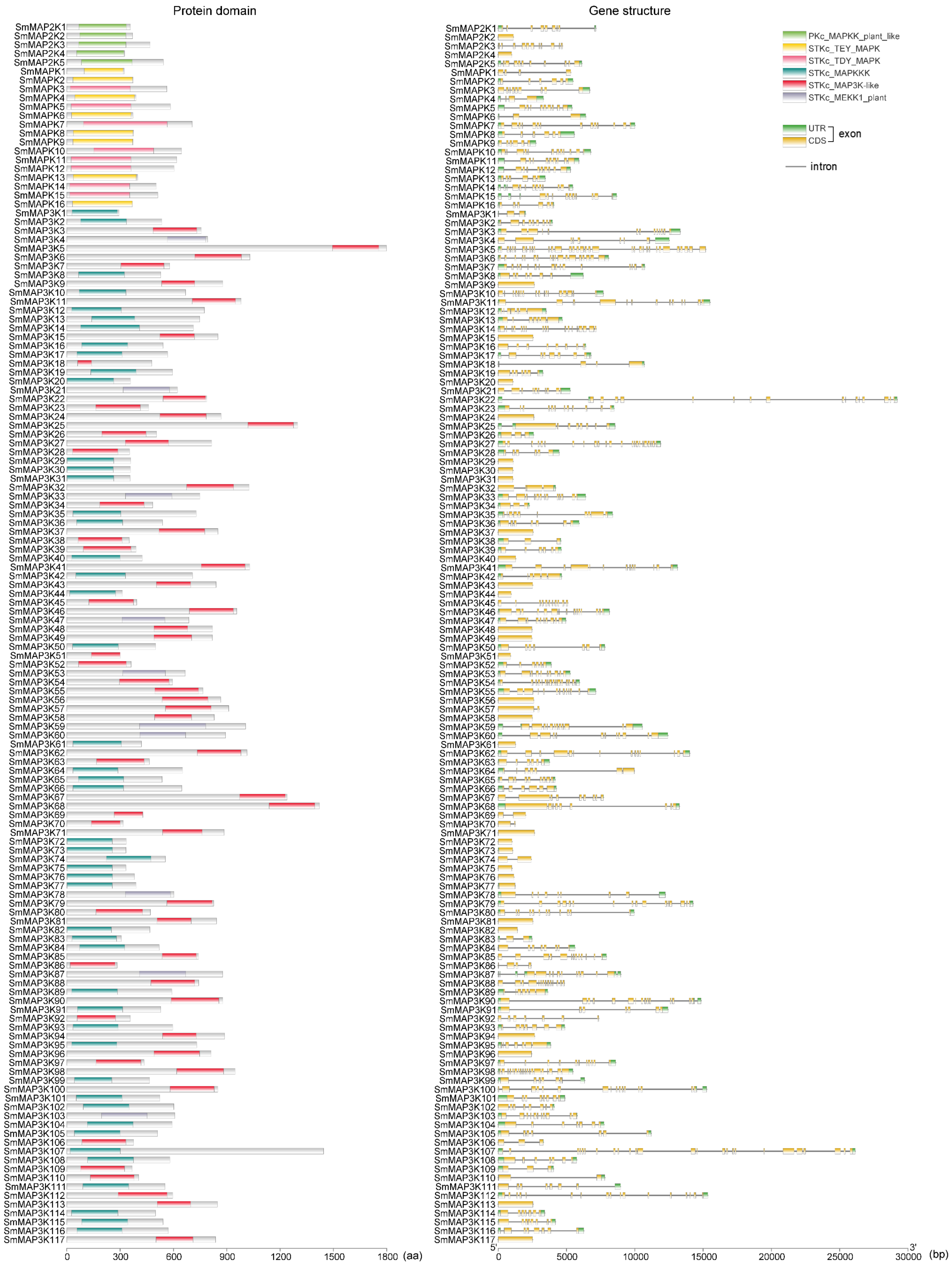

Figure S2 Gene structure and protein domain analysis of the eggplant MAPK family. Cis-acting elements involved in diverse biological processes are mapped to their positions within the promoter. Scale: base pair (bp).

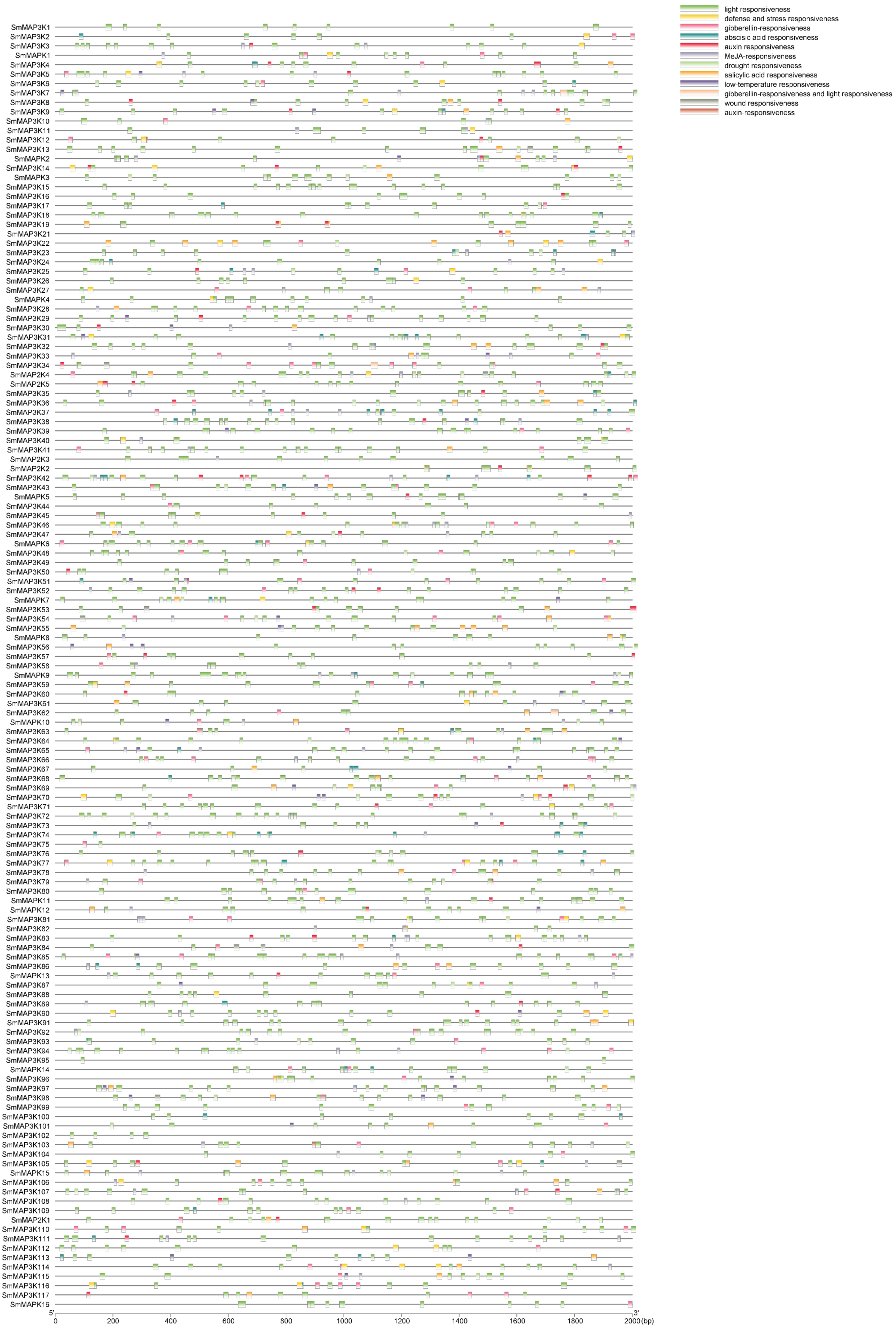

Figure S3 Expression level disease resistance marker genes of eggplant in response to *Botrytis cinerea* infection. Disease-resistance marker genes were identified by alignment with the *Solanaceous* plant tomato, and the specific numbers of expression changes are indicated in the heatmap.

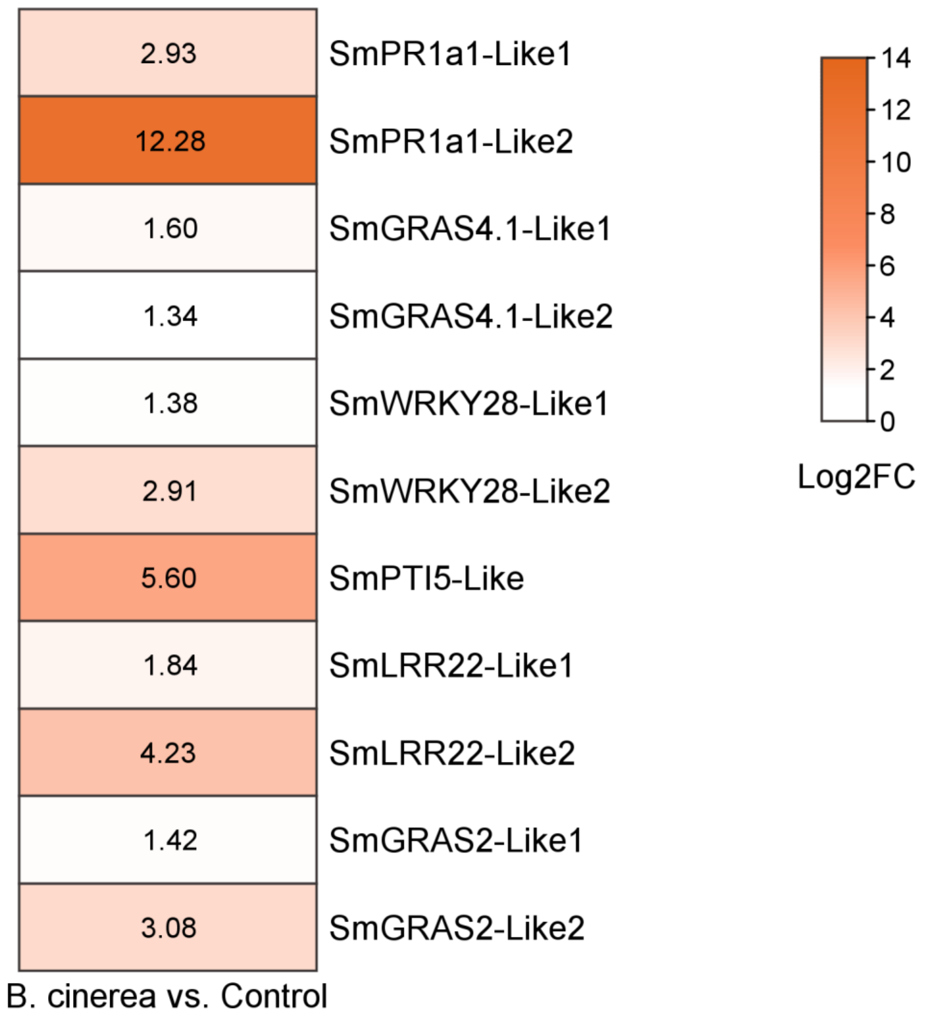

Figure S4 Expression level analysis of the eggplant MAPK family genes in response to *Botrytis cinerea* infection. The significant differential expression between groups is determined with DESeq2 (negative-binomial model, FDR < 0.01, |log2FC| ≥ 1). Genes with undetectable expression are shown in gray.

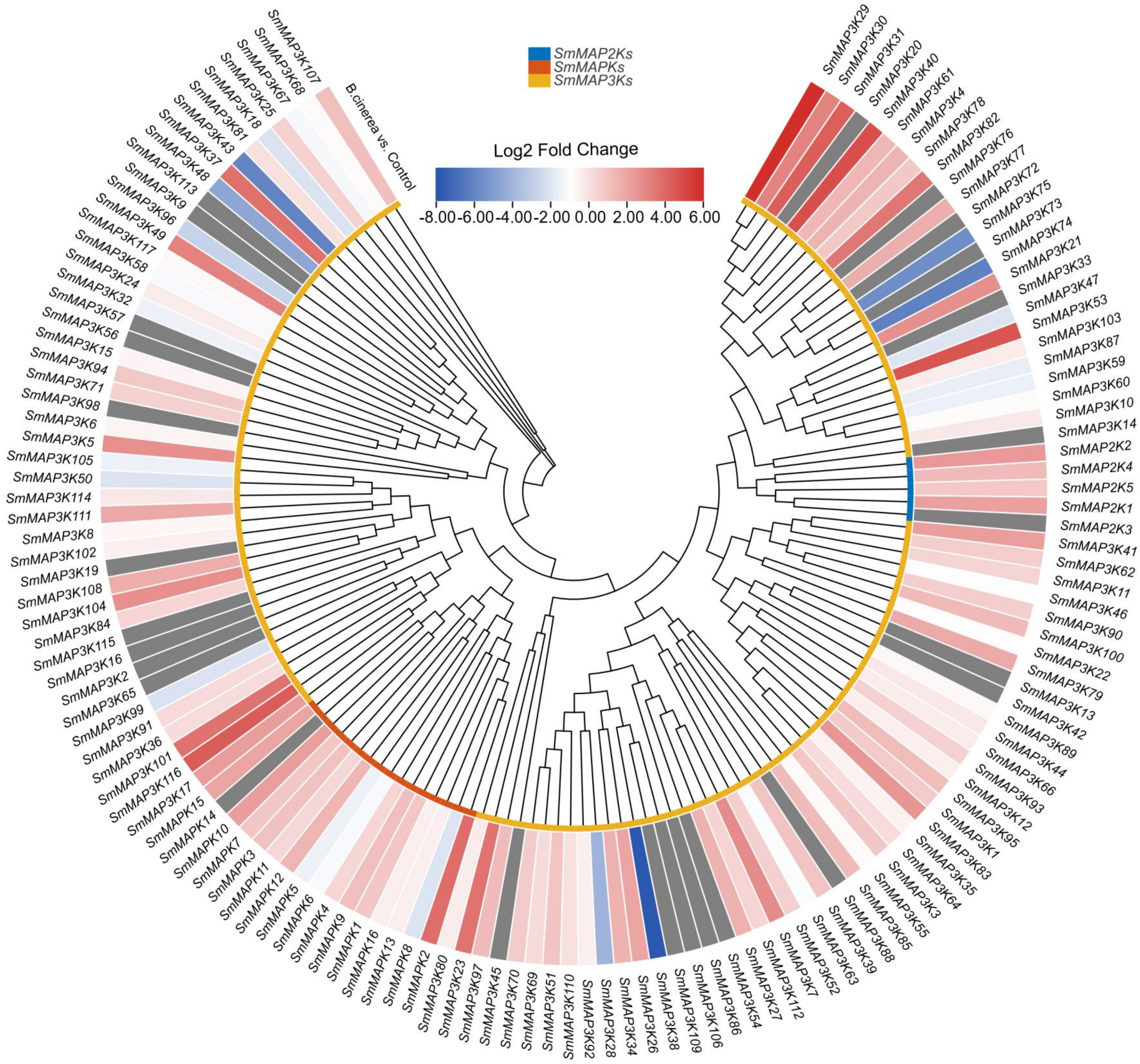

Supplement: Supplementary file 2 [file DataSheet1.pdf]
